# New remote cerebral microbleeds in acute ischemic stroke: an analysis of the randomized, placebo-controlled WAKE-UP trial

**DOI:** 10.1007/s00415-022-11175-y

**Published:** 2022-05-19

**Authors:** Tim Bastian Braemswig, Jan Vynckier, Märit Jensen, Florent Boutitie, Ivana Galinovic, Claus Z. Simonsen, Bastian Cheng, Tae-Hee Cho, Jan F. Scheitz, Jens Fiehler, Josep Puig, Vincent Thijs, Jochen B. Fiebach, Keith W. Muir, Norbert Nighoghossian, Martin Ebinger, Salvador Pedraza, Götz Thomalla, Christian Gerloff, Matthias Endres, Robin Lemmens, Ludwig Schlemm, Christian H. Nolte, on behalf of the WAKE-UP Investigators

**Affiliations:** 1 Klinik und Hochschulambulanz für Neurologie, Charité – Universitätsmedizin Berlin, Freie Universität Berlin and Humboldt-Universität Zu Berlin, Berlin, Germany; 2 Berlin Institute of Health (BIH), Charité – Universitätsmedizin Berlin, Berlin, Germany; 3 Center for Stroke Research Berlin (CSB), Charité – Universitätsmedizin Berlin, Freie Universität Berlin and Humboldt-Universität zu Berlin, Berlin, Germany; 4 Neurology Department, Head of Stroke Unit, Moorselbaan 164, 9300 Aalst, Belgium; 5 Klinik und Poliklinik Für Neurologie, Kopf- und Neurozentrum, Universitätsklinikum Hamburg–Eppendorf, Hamburg, Germany; 6 Service de Biostatistique, Hospices Civils de Lyon, Lyon, France; 7 UMR 5558, Laboratoire de Biométrie et Biologie Evolutive, Université Lyon 1 and Centre National de la Recherche Scientifique, Equipe Biostatistique-Santé, Villeurbanne, France; 8 Department of Neurology, Aarhus University Hospital, Aarhus, Denmark; 9 Department of Stroke Medicine, Université Claude Bernard Lyon 1, and Hospices Civils de Lyon, Lyon, France; 10 German Center for Cardiovascular Research (DZHK), Partner Site Berlin, Berlin, Germany; 11 Department of Diagnostic and Interventional Neuroradiology, University Medical Center Hamburg–Eppendorf, Hamburg, Germany; 12 Doctor Josep Trueta University Hospital, Department of Radiology (IDI), Girona Biomedical Research Institute (IDIBGI), Girona, Spain; 13 Department of Neurology, Austin Health, Heidelberg, Australia; 14 Institute of Neuroscience and Psychology, University of Glasgow, Glasgow, UK; 15 Department of Neurology, Medical Park Berlin Humboldtmühle, Berlin, Germany; 16 German Center for Neurodegenerative Diseases (DZNE), Partner Site Berlin, Berlin, Germany; 17 ExcellenceCluster NeuroCure, Charité-Universitätsmedizin Berlin, Berlin, Germany; 18 Department of Neurology, University Hospitals Leuven, Leuven, Belgium; 19 Department of Neurosciences, Experimental Neurology, KU Louvain–University of Leuven, Leuven, Belgium; 20 VIB-KU Leuven Center for Brain and Disease Research, Laboratory of Neurobiology, Leuven, Belgium; 21 Stroke Division, Florey Institute of Neuroscience and Mental Health, University of Melbourne, Melbourne, Australia

Dear Sirs,

Cerebral microbleeds (CMB), detected as hypointense foci on blood-sensitive magnet resonance imaging (MRI), are a marker of cerebral small vessel disease [1]. Neuropathological examinations revealed that a subset of CMBs contain intact erythrocytes, indicating recent microhemorrhage [1]. In a prior observational study, new CMBs occurred in 4% of acute ischemic stroke (AIS) patients after receiving intravenous thrombolysis (IVT) with alteplase. New CMBs were associated with an increased risk for post-IVT intracerebral hemorrhage [2].

Here, we took the unique opportunity to assess the risk of IVT for the occurrence of new CMBs in AIS patients who had been randomly assigned to either alteplase (0.9 mg/kg) or placebo within the WAKE-UP trial (Efficacy and Safety of MRI-Based Thrombolysis in Wake-Up Stroke, ClinicalTrials.gov: NCT01525290)[3].

Eligible patients for this exploratory analysis had received two cerebral MRI examinations—one before and one after treatment (alteplase or placebo)—with the same field strength (i.e., 1.5 Tesla or 3 Tesla) and the same blood-sensitive MRI sequence (i.e., susceptibility weighted imaging (SWI) or T2*/gradient-recalled echo (GRE)). Parameters of the blood-sensitive MRI sequences varied according to center and manufacturer with slice thickness ranging from 1.5 to 7 mm, echo time (TE) from 11 to 51 ms, and repetition time (TR) from 460 to 2460 ms [4].

The CMBs were defined, according to consensus recommendations, as small (up to 10 mm in diameter), round or oval hypointense lesions with associated blooming seen on blood-sensitive MRI sequences [1]. The CMBs were rated by three raters (TBB, JV, LS) blinded to clinical information (with a very good inter-rater reliability as described previously [4]). New CMBs were categorized according to their distribution as lobar, deep (i.e., basal ganglia, thalamus) or infratentorial (i.e., brainstem, cerebellum) [4]. Newly appearing CMBs in the acute infarcted area, as seen on diffusion-weighted imaging, were not classified as new CMBs [2, 4].

Intracranial hemorrhage (ICH) was assessed on follow-up imaging 22 to 36 h after treatment.

The primary outcome was the proportion of patients with new CMBs on follow-up imaging (in the alteplase and placebo group). Secondary outcomes included symptomatic ICH according to the protocol of the Safe Implementation of Thrombolysis in Stroke–Monitoring Study (SITS-MOST) [5] and degree of disability, quantified on the modified Rankin Scale (mRS) 90 days after treatment (excellent outcome: mRS ≤ 1, severe dependency or death: mRS 4–6) [3].

Of 503 patients included in the WAKE-UP trial, 350 patients were available for final analysis (175 with alteplase, 175 with placebo; 129 patients were not sequentially examined on the same MRI scanners, image quality of the blood-sensitive MRI sequence was too poor for analysis in 24 patients). Patients included in the final analysis did not differ regarding age and sex compared to excluded patients (mean age: 64.7 [patients included, standard deviation [SD]: 11.8] vs. 66.3 [patients excluded, SD: 10.8], *p* = 0.15, *t* test; female: 120/350 [34.3%] vs. 58/153 [37.9%], *p* = 0.50, chi-squared test). However, there were fewer ICH in patients included in the final analysis (76/350 [22%] vs. 47/146 [32%]; *p* = 0.02, chi-squared test).

New CMBs occurred in four patients. Noteworthy, all patients with new CMBs had received alteplase resulting in a rate of 2.3% (4/175 patients; 95% confidence interval: 0.6–5.8%). Three patients had one new CMB and one patient had two new CMBs. Distribution of new CMBs was strictly lobar in all four patients (a pattern suggestive of underlying cerebral amyloid angiopathy [1, 6]) and preexisting CMBs were present in all patients with new CMBs. One patient with new CMBs had a severe stroke with an NIHSS score of 20 on admission. Any ICH occurred in two patients with new CMBs (hemorrhagic transformation of the infarcted brain tissue (HI2, Class 1b) in one patient and intracerebral hemorrhage within and beyond the infarcted brain tissue (PH2, Class 2) in the other patient [7]). One new CMB occurred contralateral to the hemorrhagic transformation (HI2, Class 1b) in one patient, two new CMBs occurred ipsilateral to the intracerebral hemorrhage (PH2, Class 2) in the other patient. Symptomatic ICH according to SITS-MOST occurred in one patient with new CMBs. Of note, of 76 patients with any ICH, 74 ICHs occurred in patients without new CMBs. At 90 days, one patient with new CMBs achieved an excellent functional outcome (mRS ≤ 1) and two patients with new CMBs had either died or had severe disability. Further details are shown in Table 1. Due to the small number of patients with new CMBs, no inferential statistical analysis was performed.

**Table 1:** Baseline characteristics and outcomes of patients with and without new cerebral microbleeds

	All (*n* = 350)	No new CMBs (*n* = 346)	New CMBs (*n* = 4)
Demographics			
Age, mean (SD)	64.7 (11.8)	64.7 (11.9)	55, 59, 74, 74^a^
Male, % (n)	230 (65.7%)	226 (65.3%)	4/4 (100%)
Risk factors, % (*n*)			
Atrial fibrillation	40 (11.6%)	40 (11.7%)	0/4 (0%)
Diabetes mellitus, type 2	50 (14.5%)	49 (14.3%)	1/4 (25%)
Arterial hypertension	183 (52.4%)	181 (52.5%)	2/4 (50%)
History of ischemic stroke	45 (12.9%)	45 (13.0%)	0/4 (0%)
On admission			
NIHSS score, median (IQR)	5 (3;8)	5 (3;8)	1, 5, 6, 20^a^
Platelet aggregation inhibitors, n (%)	108 (30.9%)	107 (30.9%)	1/4 (25%)
Statins, *n* (%)	108 (30.9%)	105 (30.3%)	3/4 (75%)
MRI characteristics, *n* (%)			
Blood-sensitive sequence			
T2*/GRE SWI	322 (92%) 28 (8%)	319 (92.2%) 27 (7.8%)	3/4 (75%) 1/4 (25%)
Field strength			
1.5 Tesla 3 Tesla	175 (50%) 175 (50%)	173 (50%) 173 (50%)	2/4 (50%) 2/4 (50%)
Time interval between treatment and follow-up MRI, hours, median (IQR)^b^	25.5 (23.5;28.3)	25.6 (23.5;28.3)	22.7, 24.1, 24.5, 29.6^a^
Pretreatment MRI findings			
CMB presence on pretreatment MRI, *n* (%)	68 (19.4%)	64 (18.5%)	4/4 (100%)
Number of CMBs on pretreatment MRI, median (IQR)	0 (0;0)	0 (0;0)	1, 1, 5, 5^a^
Number of CMBs on pretreatment MRI, *n* (%)			
0 1 2–4 ≥ 5	282 (80.6%) 32 (9.1%) 23 (6.6%) 13 (3.7)	282 (81.5%) 30 (8.7%) 23 (6.6%) 11 (3.2%)	0/4 (0%) 2/4 (50%) 0/4 (0%) 2/4 (50%)
CMBs with a strictly lobar distribution on pretreatment MRI, *n* (%)	32/68 (47.1%)	29/64 (45.3%)	3/4 (75%)
CMBs with a mixed or strictly deep distribution on pretreatment MRI, *n* (%)	30/68 (44.1%)	30/64 (46.9%)	0/4 (0%)
Diffusion-weighted imaging lesion volume at baseline, mL, median (IQR)^c^	1.9 (0.7;7.1)	1.9 (0.7;7.1)	0.7, 2.6, 6.3, 18.0
Lacunar infarcts, *n* (%)	82 (23.4%)	82 (23.7%)	0/4 (0%)
Treatment, *n* (%)			
Alteplase Placebo	175 (50%) 175 (50%)	171 (49.4%) 175 (50.6%)	4/4 (100%) 0/4 (0%)
Outcomes			
Any intracranial hemorrhage after treatment, *n* (%)	76 (21.7%)	74 (21.4%)	2/4 (50%)
Symptomatic intracranial hemorrhage after treatment (SITS-MOST), *n* (%)	3 (0.9%)	2 (0.6%)	1/4 (25%)
mRS at 90 days, median (IQR)	1 (1;3)	1 (1;3)	1, 2, 5, 5^a^
Excellent outcome (mRS ≤ 1) at 90 days, n (%)	176 (50.9%)	175 (51.2%)	1/4 (25%)
Severe dependency or death (mRS 4–6) at 90 days, *n* (%)	42 (12.1%)	40 (11.7%)	2/4 (50%)

*CMBs* cerebral microbleeds, *GRE* gradient-recalled echo, *IQR* interquartile range, *MRI* magnetic resonance imaging, *mRS* modified Rankin Scale, *NIHSS* National Institutes of Health Stroke Scale, *SD* standard deviation, *SITS-MOST* Safe Implementation of Thrombolysis in Stroke–Monitoring Study, and *SWI* susceptibility weighted imaging

^a^Due to the small number of outcomes, quantitative variables (age, NIHSS score, time interval between treatment and follow-up MRI, number of CMBs on pretreatment MRI, diffusion-weighted imaging lesion volume at baseline, mRS at 90 days) are reported as single values in patients with new CMBs (*n* = 4)

^b^The variable time interval between treatment and follow-up MRI was known in 345 patients without new CMBs

^c^The variable diffusion-weighted imaging lesion volume at baseline was known in 343 patients without new CMBs

In this exploratory analysis of the randomized, placebo-controlled WAKE-UP trial, new CMBs occurred exclusively after receiving alteplase.

In the recently published THAWS trial (THrombolysis for Acute Wake-up and Unclear-onset Strokes With Alteplase at 0.6 mg/kg Trial [conducted in Japan]), new CMBs occurred in seven out of 113 AIS patients and were associated with poor neurological outcome [8]. In both study cohorts (WAKE-UP and THAWS) new CMBs occurred only in AIS patients receiving alteplase (as opposed to placebo).

The small number of outcomes, the lack of an inferential statistical analysis, a possible selection bias (ICH occurred more often in patients excluded from this analysis) as well as varying parameters of the blood-sensitive MRI sequences (e.g. slice thickness) in the different centers have to be taken into account when interpreting our findings. However, by comparing to placebo we were able to assess the effect of alteplase for the occurrence of new CMBs, thereby extending the knowledge of previous observational studies. Considering our results as well as the above-mentioned results from the THAWS trial, there is now accumulating evidence that alteplase contributes to the development of new CMBs in AIS patients. Although new CMBs occur rarely, our data call for further studies to determine if these new CMBs after receiving alteplase increase the risk of poor neurological outcome in AIS patients.

## Abbreviations

AIS: Acute ischemic stroke
CAA: Cerebral amyloid angiopathy
CMB: Cerebral microbleed
ICH: Intracranial hemorrhage
IVT: Intravenous thrombolysis
MRI: Magnetic resonance imaging
mRS: Modified Rankin Scale
NIHSS: National Institutes of Health Stroke Scale
SD: Standard deviation
